# Strahlenschutzrechtliche Verfahren im Kontext des neuen Medizinforschungsgesetzes: Was ändert sich, was bleibt? Ein gemeinsamer Überblick von der Deutschen Gesellschaft für Radioonkologie (DEGRO) und dem Bundesamt für Strahlenschutz (BfS)

**DOI:** 10.1007/s00066-025-02488-8

**Published:** 2025-11-20

**Authors:** Elena Sperk, Matthias Habeck, Felix Giani, Dirk Vordermark, Mechthild Krause, Stefan Rieken, Ursula Nestle, Bastian Breustedt

**Affiliations:** 1 Mannheim Cancer Center, Universitätsmedizin Mannheim, Medizinische Fakultät Mannheim, Universität Heidelberg, Theodor-Kutzer-Ufer 1–3, 67165 Mannheim, Deutschland; 2 Bundesamt für Strahlenschutz, Willy-Brandt-Straße 5, 38226 Salzgitter, Deutschland; 3 Universitätsklinik und Poliklinik für Strahlentherapie, Universitätsklinikum Halle (Saale), Ernst-Grube-Straße 40, 06120 Halle (Saale), Deutschland; 4 Klinik für Strahlentherapie, Medizinische Fakultät und Universitätsklinikum C.G. Carus, TU Dresden, Fetscherstraße 74, 01307 Dresden, Deutschland; 5 Nationales Zentrum für Tumorerkrankungen (NCT) Dresden, DKFZ Heidelberg, Universitätsklinikum und Medizinische Fakultät C.G. Carus Dresden sowie Helmholtz-Zentrum Dresden-Rossendorf, Dresden, Deutschland; 6 Klinik für Strahlentherapie und Radioonkologie, Universitätsmedizin Göttingen, Georg-August-Universität, Robert-Koch-Straße 40, 37075 Göttingen, Deutschland; 7 Klinik für Strahlentherapie und Radioonkologie, Kliniken Maria Hilf, Viersener Straße 450, 41063 Mönchengladbach, Deutschland

**Keywords:** StrSchG - Strahlenschutzgesetz, MFG - Medizinforschungsgesetz, Strahlenanwendung in der Forschung, Begleitdiagnostik, Klinische Studie

## Abstract

Durch das Medizinforschungsgesetz sind viele Aspekte auch im Genehmigungs- und Anzeigeverfahren für studienbedingte Strahlenanwendungen angepasst worden. Dieser Artikel soll einen Überblick über die neue Situation geben und den Forschenden, insbesondere im Bereich der Radioonkologie, als Wegweiser für die medizinisch-rechtliche Planung von Studienprojekten dienen.

## Hintergrund

Der Einsatz diagnostischer und therapeutischer Strahlenanwendungen in der Medizin ist aus der täglichen Routine und der klinischen Forschung nicht mehr wegzudenken. Bei Patienten mit onkologischen Erkrankungen stellt die radiologische und nuklearmedizinische Bildgebung die zentrale Grundlage für personalisierte interdisziplinäre Therapieentscheidungen dar. Zudem sind therapeutische Strahlenanwendungen in der Nuklearmedizin und Radioonkologie an fast der Hälfte aller Krebsheilungen beteiligt [1].

Seit vielen Jahrzehnten sind allerdings auch die Risiken ionisierender Strahlung bekannt. Bereits bei einer diagnostischen Strahlenexposition onkologischer Patienten muss von einem mit der Untersuchungshäufigkeit steigenden Zweitmalignomrisiko ausgegangen werden – in Anbetracht der zunehmenden Heilungschancen ein ernstzunehmendes Thema. Bei therapeutischen Dosen kommen akute und späte Strahlenfolgen an den bestrahlten gesunden Geweben hinzu, die sich erheblich auf Prognose und Lebensqualität auswirken können.

So gilt es immer, den Nutzen diagnostischer und therapeutischer Strahlenanwendungen gegen ihre möglichen Risiken abzuwägen. Im Rahmen der klinischen Routine geschieht diese Abwägung auf einer patientenindividuellen Ebene durch fachkundige Ärzte beim Stellen der rechtfertigenden Indikation. Geschieht eine Strahlenanwendung im Rahmen der Forschung, erfolgt die auch hier gebotene Nutzen-Risiko-Abwägung hingegen auf der Ebene des Studienkollektivs, dessen genaue Zusammensetzung durch die Ein- und Ausschlusskriterien des Studienprotokolls bestimmt wird.

Der erwartbare Nutzen von studienbedingten Strahlenanwendungen besteht vor allem in einem studienbedingten Erkenntnisgewinn und stellt einen potenziellen Mehrwert für die Allgemeinheit dar. Typischerweise besteht für die an der Studie teilnehmenden einzelnen Personen jedoch eine gewisse Unsicherheit hinsichtlich ihres individuellen Nutzens und Risikos.

**Abb. 1:**
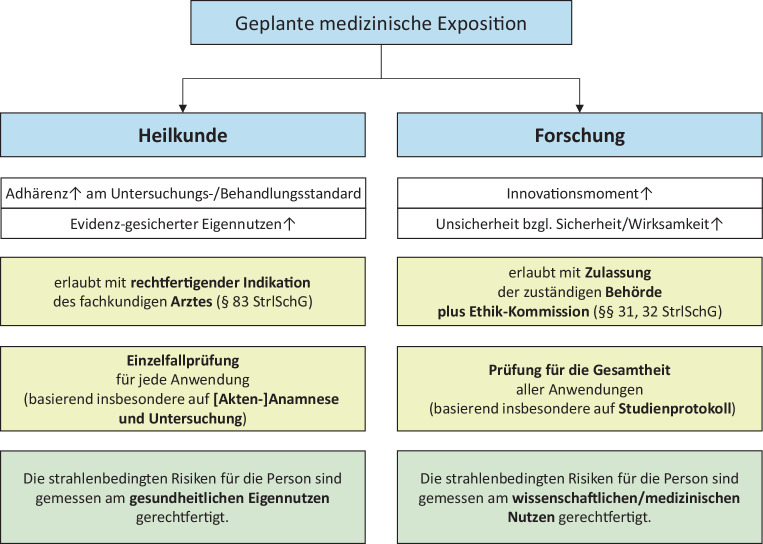
Gegenüberstellung der beiden wichtigsten Anwendungsbereiche für ionisierende Strahlung & radioaktive Stoffe am Menschen

Es ist daher in der klinischen Forschung wichtig zu entscheiden, ob die Durchführung einer Strahlenanwendung der regulären Krankenversorgung entspricht („Heilkunde“) oder in den Bereich der Forschung gehört (Abb. 1). Dieser Unterschied sowie mögliche Zielkonflikte zwischen den beiden Bereichen sind der Hintergrund für die hier geltenden Regelungen der Richtlinie 2013/59/Euratom [2]. Diese schreibt vor, dass Projekte mit medizinischen Strahlenanwendungen zu Forschungszwecken vorab von einer externen Stelle geprüft (ggf. genehmigt) werden müssen, wenn diese nicht der regulären Krankenversorgung entsprechen. Somit müssen sich Studientreibende in der klinischen Forschung nicht erst bei der individuellen Therapieplanung, sondern bereits im Rahmen der Studienplanung mit Fragen des Strahlenschutzes auseinandersetzen. Dies kann vergleichsweise einfach sein, wenn z. B. eine Routinebildgebung zur Kontrolle des Behandlungserfolgs genügt. Es kann aber auch sehr komplex sein, wie z. B. bei der neuartigen FLASH-Radiotherapie [3], wo vielleicht manche Grundsätze der Strahlenbiologie neu justiert werden müssen, bevor das Potenzial der Methode voll ausgeschöpft werden kann.

Die nationale Umsetzung des einschlägigen Artikels 55 der EURATOM-Richtlinie ist formulierungsbedingt in Europa heterogen erfolgt: Der einschlägige Absatz 2 Buchstabe e lässt offen, ob die Zuständigkeit für diese Prüfung seitens des nationalen Gesetzgebers in die Hände einer Ethikkommission „und/oder“ einer Behörde gelegt wird.

## Grundzüge und Entwicklung der Regelwerke in Deutschland

Forschungsvorhaben, bei denen sich eine Strahlenanwendung im Rahmen der regulären Heilkunde (Abb. 1) bewegt, benötigen keine strahlenschutzrechtliche Vorabprüfung, selbst wenn die zu erhebenden Daten im Nachhinein wissenschaftlich ausgewertet werden sollen. Die Strahlenschutzbewertung erfolgt hier über die ohnehin erfolgende ärztliche Indikationsstellung.

**Abb. 2:**
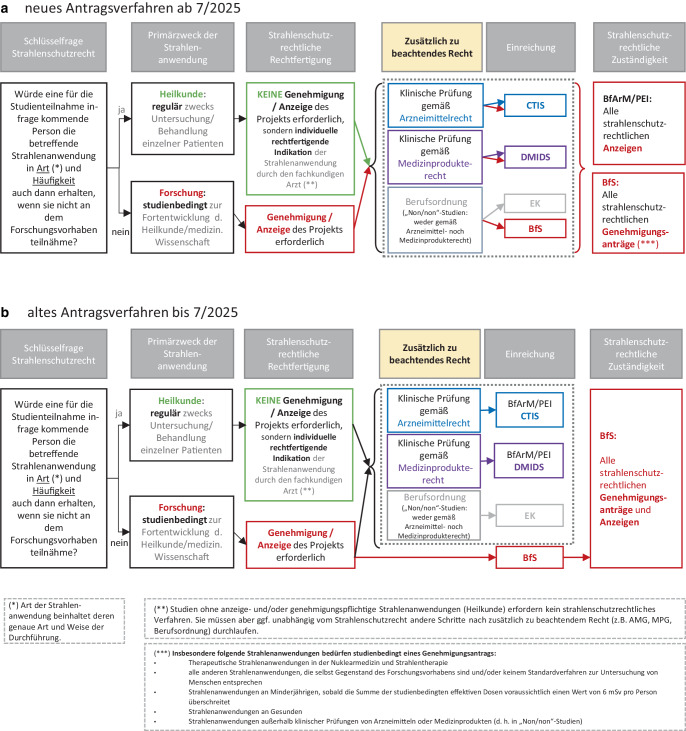
Workflow der Anzeige- und/oder Genehmigungsverfahren nach Strahlenschutzrecht, **a** neues Antragsverfahren, **b** altes Antragsverfahren

Sobald hingegen ionisierende Strahlung zum Zweck der Forschung eingesetzt wird, ist – neben anderweitigen, insbesondere im Arzneimittel‑, Medizinprodukte- und Berufsrecht verankerten Prüfungen durch Ethikkommissionen und Bundesoberbehörden im Geschäftsbereich des Bundesministeriums für Gesundheit (BMG) wie das Bundesinstitut für Arzneimittel und Medizinprodukte (BfArM) und das Paul-Ehrlich-Institut (PEI) – im Sinne einer behördlichen Vorabkontrolle [2] ein gesondertes strahlenschutzrechtliches Bewertungsverfahren erforderlich. Das deutsche Strahlenschutzrecht unterscheidet hierfür heute grundsätzlich zwei Verfahren unterschiedlicher Prüftiefe (Abb. 2):

- Anzeigeverfahren: z. B. für standardgemäße Begleitdiagnostik
- Genehmigungsverfahren: z. B. für Strahlenanwendungen, die selbst Forschungsgegenstand sind

Bis Anfang der 2000er-Jahre lag die Zuständigkeit für das Genehmigungsverfahren bei den Landesbehörden, die heute lediglich noch als Aufsichtsbehörden fungieren. Aufgrund zunehmender fachlicher Anforderungen wurde diese Aufgabe ab 2001 auf das BfS übertragen [4]. 2011 wurde ein vereinfachtes Genehmigungsverfahren für sogenannte Begleitdiagnostik wie CT-Untersuchungen zur Therapiebeurteilung bei Erwachsenen eingeführt [5]. Mit dem Inkrafttreten des Strahlenschutzgesetzes (StrlSchG) 2018 [6] wurden erstmals gesetzliche Fristen eingeführt und das BfS personell so gestärkt, dass die o. g. Prüfungen fachgerecht und zuverlässig durchführt werden konnten. 

Gleichzeitig wurde das vereinfachte Genehmigungsverfahren in ein reines Anzeigeverfahren überführt [5]. 

In der Praxis führte jedoch die fehlende Abstimmung zwischen europäischem Strahlenschutzrecht und anderen rechtlichen Regelungen wie der Verordnung (EU) Nr. 536/2014 über klinische Prüfungen mit Humanarzneimitteln („Clinical Trial Regulation“ [CTR]; [7]) sowie der Verordnung (EU) Nr. 2017/745 über Medizinprodukte („Medical Device Regulation“ [MDR]; [8]) immer noch zu Bürokratie im Sinne von ansonsten entbehrlichem Mehraufwand. Dies hatte vielfältige Gründe, wie nicht vollständig parallelisierte Verfahren, bei zwei häufig zu kombinierenden Verfahrensarten teilweise redundante Antragsformulare, die sprachlich stark an den rechtlichen Vorgaben orientiert und für Nichtjuristen schwer verständlich waren, sowie die Notwendigkeit aufwendiger Paraphrasierungen (Umarbeitung einschlägiger Textpassagen) englischsprachiger Studienpläne, welche die für die strahlenschutzrechtliche Prüfung wichtigen Aspekte häufig selbst nicht hinreichend abbilden. Fehlende Erfahrung bei Sponsoren oder Clinical-research-Organisationen (CRO) mit dem Strahlenschutzrecht und mit den Eigenheiten medizinischer Strahlenanwendungen führten zu Nachfragen der Genehmigungsbehörde und Verzögerungen im Verfahrensablauf. Dies bewirkte, dass strahlenschutzrechtliche Verfahren in klinischen Studien als sehr aufwendig wahrgenommen und daher oft vermieden wurden, mit zwei negativen Folgen: Nachteile sowohl für den Strahlen‑/Patientenschutz als auch für die strahlenbasierte Forschung in Deutschland im Vergleich zu anderen Bereichen wie der Systemtherapie oder der Chirurgie.

## Gelegenheit zur Neuregelung

Aus der seitens BfS 2023 beim AKEK (Arbeitskreis medizinischer Ethik-Kommissionen in der Bundesrepublik Deutschland e. V.) eruierten Bereitschaft zur Übernahme der Rechtfertigungsprüfung im Anzeigeverfahren sowie der Pharmastrategie der Bundesregierung ergab sich 2024 die Chance zur Neuordnung der strahlenschutzrechtlichen Verfahren durch das neu zu erarbeitende Medizinforschungsgesetz (MFG; [8]). Dabei wurde berücksichtigt, dass es zwar sicherlich sehr im Sinne von Forschung und Gesellschaft ist, den Strahlenschutz zu wahren, dass es aber nicht nachhaltig ist, wenn dafür im EU-Kontext vergleichsweise aufwendige nationale Verfahren zu einem Wettbewerbsnachteil für die Forschung in Deutschland führen. Daher wurden Forderungen der Forschenden aufgenommen, eine weitere Beschleunigung und Harmonisierung der herausfordernden und zeitlich sowie personell als aufwendig wahrgenommenen, teilweise redundanten Prozesse zu erreichen. Gleichzeitig sollte aber der Schutzgedanke der europäischen und nationalen Regelwerke nicht vernachlässigt werden.

**Abb. 3:**
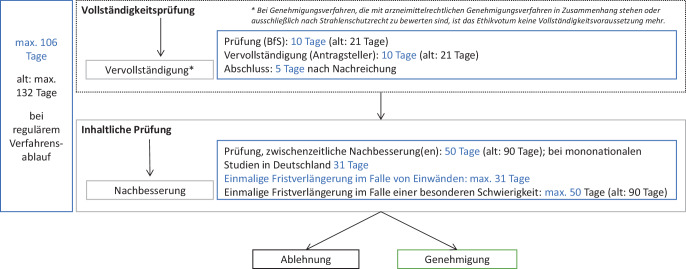
Alte und (in *Blau*) neue Fristen (ab 1. Juli 2025) im BfS-Genehmigungsverfahren

Im Zuge des MFG wurde daher das StrlSchG angepasst, um eine Neuordnung und Beschleunigung der Antragsverfahren zu ermöglichen. So wurden beispielsweise die im Gesetz vorgesehenen Verfahrensfristen für Anträge gekürzt (siehe Abb. 3), die Einreichungsvorgänge harmonisiert und die strahlenschutzrechtlichen Antrags- und Anzeigeformulare grundlegend überarbeitet. Zur Beschleunigung und Vereinfachung der Verfahren dienen auch juristische „Fiktionen“: Bereits seit 2018 gibt es im StrlSchG die sogenannte Genehmigungsfiktion. Das bedeutet: Wenn die Behörde innerhalb einer gesetzlich festgelegten Frist nicht über einen Antrag entscheidet, gilt dieser automatisch als genehmigt. Diese Regelung schützt Antragstellende vor unnötigen Verzögerungen und sorgt für mehr Planungssicherheit. Im Zuge der weiteren Modernisierung des Strahlenschutzrechts wurde nun eine zweite Regelung eingeführt: die Fiktion der Antragsrücknahme. Auch sie dient der Beschleunigung von Verfahren, allerdings in umgekehrter Richtung: Wenn Antragstellende Rückfragen oder Nachforderungen der Behörde nicht innerhalb einer festgelegten Frist beantworten, gilt der Antrag automatisch als zurückgezogen. Somit besteht für beide Seiten (Antragstellende und Genehmigungsbehörde) ein gemeinsames Interesse, die Verfahren zügig und fristgerecht durchzuführen.

Insgesamt bleiben also bei den strahlenschutzrechtlichen Verfahren einige Regelungen bestehen, manches wurde überarbeitet und manche Abläufe wurden komplett neugestaltet. Im Folgenden werden einige besonders praxisrelevante Aspekte der neuen Situation beleuchtet.

## Aktuelle Abläufe und Regelungen nach der Umsetzung des MFG (seit dem 01.07.2025)

### Das bleibt

*Heilkunde vs. Forschung:*

Unverändert bleibt die Tatsache, dass Forschungsvorhaben, bei denen Strahlenanwendungen am Menschen in Art und Häufigkeit ausschließlich im Rahmen der regulären Krankenversorgung erfolgen („*Heilkunde*“) *keine* strahlenschutzrechtliche behördliche Vorabkontrolle erfordern, auch wenn die mit ihnen zu gewinnenden Daten wissenschaftlich ausgewertet werden sollen (Abb. 1).

Bei der manchmal nicht trivialen Abwägung, ob es sich bei einzelnen Strahlenanwendungen in klinischen Studien um Heilkunde oder um Forschung handelt, leistet weiterhin das Expertengremium der DEGRO [9] beratende Unterstützung. Wie gehabt liegt die Verantwortung der Entscheidung für oder gegen die Einreichung eines Projekts aber letztlich bei der Studienleitung und insbesondere bei den fachkundigen Ärztinnen und Ärzten der teilnehmenden Studienzentren, da bei nicht anzeige- oder genehmigungspflichtigen Strahlenanwendungen in jedem individuellen Anwendungsfall eine rechtfertigende Indikation vorliegen muss. Für die Qualitätssicherung bei dieser Entscheidungsfindung und der in diesen Fällen zu stellenden rechtfertigenden Indikationen sind im Nachhinein die von den Aufsichtsbehörden benannten ärztlichen Stellen zuständig.

### Das bleibt

*Zuständigkeit für ***Genehmigungsverfahren** *(z.* *B. Therapie) weiterhin beim BfS.*

### Das ist neu

*Kriterien für die Genehmigungsbedürftigkeit studienbedingter Strahlenanwendungen.*

Nach Inkrafttreten der Änderungen des StrlSchG durch das MFG lassen sich im Rahmen von Forschungsprojekten die folgenden *fünf* Kategorien von Strahlenanwendungen unterscheiden, für die eine Genehmigung gemäß § 31 StrlSchG durch das BfS als zuständige Behörde erforderlich ist, *wenn studienbedingt in Art oder Häufigkeit vom sonst Üblichen abgewichen wird* (außerhalb der regulären Krankenversorgung befindlicher Anwendungsbereich).

- Therapeutische Strahlenanwendungen in der Nuklearmedizin und Strahlentherapie
- Alle anderen Strahlenanwendungen, die selbst Gegenstand des Forschungsvorhabens sind und/oder keinem Standardverfahren zur Untersuchung von Menschen entsprechen
- Strahlenanwendungen an Minderjährigen, sobald die Summe der studienbedingten effektiven Dosen voraussichtlich einen Wert von 6 mSv pro Person überschreitet
- Strahlenanwendungen an Gesunden
- Strahlenanwendungen in einem Forschungsvorhaben, das jenseits strahlenschutzrechtlicher Aspekte ausschließlich nach Berufsordnung von einer Ethikkommission zu prüfen ist, bei dem es sich also um *keine* klinische Prüfung gemäß Medizinprodukterecht-Durchführungsgesetz (MPDG) oder Arzneimittelgesetz (AMG) handelt, im Rahmen von sogenannten „freien“ oder „Non/non“-Studien.

Sobald ein Forschungsvorhaben mindestens eine genehmigungsbedürftige Strahlenanwendung vorsieht, sind alle studienbedingten Strahlenanwendungen in diesem Forschungsvorhaben genehmigungsbedürftig: Es gibt keine strahlenschutzrechtlichen Kombinationsverfahren aus Genehmigungs- und Anzeigeverfahren mehr.

### Das ist neu

*Zuständigkeit für die strahlenhygienische Prüfung im ***Anzeigeverfahren** *(z.* *B. Begleitdiagnostik) bei BfArM, PEI und den Ethikkommissionen.*

Mit den Änderungen des StrlSchG durch das MFG ist mit dem 01.07.2025 die Zuständigkeit für die strahlenschutzrechtliche Prüfung im Anzeigeverfahren auf die Ethikkommissionen übergegangen, die auch zuvor die Risiken diagnostischer Strahlenanwendungen bereits in ihre umfassendere Prüfung der ethischen Vertretbarkeit einbezogen hatten. Für den Bereich der strahlenhygienisch weniger bedeutsamen Begleitdiagnostik beendet das MFG somit die bisher in Teilen praktizierte Doppelprüfung strahlenhygienischer Risiken durch das BfS als zuständige Bundesoberbehörde und die Ethikkommissionen. Die formale Verfahrensführung obliegt im Anzeigeverfahren zukünftig einer der beiden arzneimittel- oder medizinprodukterechtlich zuständigen Bundesoberbehörden BfArM oder PEI. Das Anzeigeverfahren ist nur zulässig für Studien, die über die EU-Plattform CTIS (Clinical Trial Information System) oder über die Plattform DMIDS (Deutsches Medizinprodukte-Informations- und Datenbanksystem) zur Prüfung durch die Behörden eingereicht werden, und damit nur für Studien mit Medikamenten oder Medizinprodukten. Für diagnostische Strahlenanwendungen, die studienbedingt in Art und Häufigkeit vom in der regulären Krankenversorgung Üblichen abweichen und nach Berufsordnung laufen („freie“ oder „Non/non“-Studien), ist direkt beim BfS ein Genehmigungsantrag zu stellen (s. oben). Innerhalb des betreffenden Genehmigungsverfahrens stuft das BfS seine Prüftiefe je nach Art der beantragten Strahlenanwendung ab. So hängt die Prüftiefe bei BfS z. B. risikoadaptiert davon ab, ob therapeutische Strahlenanwendungen mit hoher Strahlenexposition oder komplexe diagnostische Anwendungen oder lediglich begleitdiagnostische Standardverfahren durchgeführt werden sollen.

### Das ist neu

**Anzeigeverfahren** *für begleitdiagnostische Strahlenanwendungen ***bei Minderjährigen** *möglich.*

Das bisherige Anzeigeverfahren wurde auf begleitdiagnostische Niedrigdosisstrahlenanwendungen (z. B. konventionelle Röntgenuntersuchungen oder Knochendichtemessungen) bei Minderjährigen erweitert. Für derartige Standardanwendungen in „klassischen“ kinderonkologischen Arzneimittelstudien wird zukünftig – anders als bisher – kein Genehmigungsverfahren mehr erforderlich, wenn die Summe der studienbedingten effektiven Dosen in einer „klassischen“ kinderonkologischen Arzneimittelstudie den Grenzwert von 6 Millisievert voraussichtlich nicht übersteigen wird.

### Das ist neu

*Überarbeitete Abläufe im* **Genehmigungsverfahren**.

Im Rahmen der Umsetzung des MFG wurden die Abläufe im *Genehmigungsverfahren* im BfS vereinfacht und überarbeitet. So wurden auch die Formblätter umfassend überarbeitet und Prozesse anwenderfreundlicher gestaltet.

Die wichtigsten Neuerungen seit dem 01.07.2025:

1. *„Single-Gate“-Ansatz für die Bündelung verschiedener Einreichungswege:* Sofern es sich bei dem jeweiligen Forschungsvorhaben um eine klinische Prüfung eines Medizinprodukts (nach Verordnung [EU] 2017/745 oder MPDG) bzw. eines Arzneimittels (nach AMG) handelt, erfolgt auch die Einreichung der Unterlagen für die strahlenschutzrechtliche Prüfung zentral über das jeweilige Portal (MPDG in DMDIS, AMG in CTIS). In allen anderen Projekten mit studienbedingter Strahlenanwendung im Rahmen der Forschung erfolgt die Einreichung direkt beim BfS (Abb. 2).
2. *Abschaffung der teilweise redundanten strahlenschutzrechtlichen „Kombiverfahren“:* Die bisher übliche Kombination aus Genehmigungs- und Anzeigeverfahren entfällt. Künftig reicht ein einziges Genehmigungsverfahren beim BfS aus, sobald eine klinische Studie mindestens eine genehmigungspflichtige Strahlenanwendung enthält. Das bedeutet: Wird z. B. der Erfolg einer genehmigungspflichtigen Strahlentherapie durch eine studienbedingte CT-Diagnostik begleitet, ist dafür kein separates Anzeigeverfahren mehr nötig. Die diagnostische Maßnahme wird im Rahmen des Gesamtantrags mitgeprüft – bei Standardverfahren in einer Prüftiefe vergleichbar mit der bisherigen Anzeige.

3. *Entfall der Notwendigkeit zur Vorlage des Ethikvotums vor der Antragstellung beim BfS:* Bisher war das Vorliegen eines Votums der Ethikkommission ein Vollständigkeitserfordernis bei der Antragstellung. Diese Anforderung wurde gelockert, sodass das Votum bei Studien nach AMG oder Berufsordnung erst später dem BfS nachgereicht werden kann. Für Medizinproduktestudien ist dies nicht möglich, da nach dem MPDG weiterhin ein sequenzieller Ablauf der Verfahren vorgesehen ist.
4. *Harmonisierung der Fristen in Relation zur CTR* (EU-Verordnung Nr. 536/2014): Die Fristen der parallel laufenden Verfahren der verschiedenen Rechtsgebiete wurden aneinander angepasst. Das führt zu einer Verkürzung im strahlenschutzrechtlichen Genehmigungsverfahren. Erstmals wurden auch dedizierte Fristen für Änderungsverfahren (kürzer als für Erstverfahren) eingeführt (Abb. 3).
5. *Implementierung einer neuen Fiktion auch aufseiten der Antragsteller (s.* *oben):* die Fiktion der Antragsrücknahme. Wenn Antragstellende Rückfragen oder Nachforderungen der Behörde nicht innerhalb einer festgelegten Frist beantworten, gilt der Antrag automatisch als zurückgezogen.

## Zusammenfassung der wichtigsten Eckpunkte strahlenschutzrechtlicher Verfahren nach Inkrafttreten der Änderungen des StrlSchG durch das neue MFG

- Die arzneimittel-, medizinprodukte- und strahlenschutzrechtlichen Genehmigungsregularien werden angesichts unterschiedlicher Grundlagen auch im europäischen Recht weiterhin unabhängig voneinander bestehen bleiben.
- Es gibt keine Änderung hinsichtlich der grundlegenden Frage, ob eine strahlenschutzrechtliche Einreichung überhaupt erforderlich ist (Abb. 1).
- Änderungen betreffen die Zuständigkeiten für das Anzeigeverfahren (formale Zuständigkeit in Studien nach AMG und MPDG zukünftig bei den beiden arzneimittelrechtlichen Bundesoberbehörden BfArM und PEI) sowie die Frage, ob ein Genehmigungsantrag oder eine Anzeige erforderlich ist.
- Vereinfachungen der Verfahren durch Einführung des „Single Gate“ (nur ein einziger Einreichungsort pro klinische Prüfung) sowie Verfahrensbeschleunigungen (Fristverkürzungen im Genehmigungsverfahren, nochmals kürzere Fristen bei Änderungsverfahren)

Insgesamt stellt das MFG eine Novellierung des StrlSchG größeren Umfangs mit Änderungen des Verfahrenszuschnitts, der Zuständigkeiten sowie der Einreichungswege dar (Tab. 1). Im Einklang mit der Richtlinie 2013/59/Euratom [2] wird die Rolle der Ethikkommissionen bei der Prüfung von diagnostischen Standardverfahren gestärkt, während das BfS sich mit seiner spezifischen strahlenmedizinischen Fachkompetenz stärker auf genehmigungsbedürftige Strahlenanwendungen (inkl. aller studienbedingten strahlentherapeutischen Anwendungen) mit Ausweitung seines Beratungsangebots bereits im Vorfeld der Antragstellung fokussieren kann.

**Tab. 1:** Tabellarische Übersicht der wichtigsten StrlSchG-Änderungen infolge des MFG

Neuerung	Erläuterung
„Single Gate“	Pro klinische Studie nur noch ein einziger Einreichungsort, entweder via CTIS, DMIDS oder per E‑Mail direkt beim BfS (Abb. 2)
Verfahrensbeschleunigung	Fristverkürzungen sowohl für Erst- als auch für Änderungsanträge, primär an den kürzeren Fristen der CTR orientiert (Abb. 3)
	Entfall des Ethikvotums als Vollständigkeitserfordernis bei der Antragstellung (mit Ausnahme von MPDG-Studien) für stärkere Parallelisierung der Verfahren bei Ethikkommissionen und BfS
	Neben bereits gegebener Genehmigungsfiktion Ergänzung einer Fiktion der Antragsrücknahme
Ausweitung des Anzeigeverfahrens	Einbeziehung von Minderjährigen, sofern 6 mSv nicht überschritten werden
Abschaffung von strahlenschutzrechtlichen „Kombiverfahren“ und sonstigen Redundanzen	Bei Anzeigen Übernahme der Verfahrensführung durch BfArM/PEI sowie der inhaltlichen Prüfung durch die Ethikkommissionen, bei Non/non-Studien einheitlich BfS-Genehmigungsverfahren
	Vereinfachung der Formblätter

Die Änderungen gelten ausschließlich für Studien, bei denen Strahlenanwendungen *studienbedingt in Art oder Häufigkeit vom sonst Üblichen abweichen*, die sich also außerhalb der regulären Krankenversorgung bewegen. Studien, die Strahlenanwendungen im Rahmen der Heilkunde anwenden, bedürfen weder einer Anzeige noch einer Genehmigung für die Strahlenanwendungen durch die Behörden.

## Sonstige Schritte parallel zur MFG-Umsetzung

*BfS-Internetauftritt*: Der BfS-Internetauftritt für den Bereich der medizinischen Forschung (https://www.bfs.de/medizinische-forschung) wurde grundlegend neu strukturiert. Dabei wurde eine neue „Orientierungshilfe zum Vorgehen bei der Einreichung“ [10] integriert, um die Klärung der Notwendigkeit eines strahlenschutzrechtlichen Verfahrens und die Wahl der richtigen Verfahrensart bestmöglich zu unterstützen. Darüber hinaus wurden die Einreichungsmodalitäten (früher „Checkliste“ zur Komplettierung der einzureichenden Unterlagen) und die FAQs (Frequently Asked Questions) im Hinblick auf die neue Rechtslage aktualisiert. Die Informationen und Formblätter auf der BfS-Homepage werden laufend aktualisiert werden, um bei der Umsetzung des MFG und nach der Sammlung erster Erfahrungen möglichst flexibel auf neu identifizierte Anforderungen reagieren und ggf. kurzfristig weitere, gebotene Erleichterungen schaffen zu können.

Die bisherigen medizinisch-wissenschaftlichen *BfS-Formblätter* wurden überarbeitet und in ein einziges Formular überführt. Im neuen Formblatt sollen verschiedene Abschnitte mit farblicher Kennzeichnung dabei helfen, nur relevante Teile auszufüllen und alle studienbedingten Strahlenanwendungen unabhängig von ihrer Art (therapeutisch/diagnostisch/begleitdiagnostisch) in nur noch einem Dokument zu bearbeiten. Die bisherige Aufteilung auf Dokumente für 4 Fallgruppen und zusätzliche Anlagen entfällt. Zusätzlich gibt es ein Formblatt für die administrativen Inhalte, in dem die bisher in verschiedenen Dokumenten abgefragten Inhalte zusammengefasst und gestrafft sind. Für die Genehmigung begleitdiagnostischer Strahlenanwendungen (Standardanwendungen) wird auf die Angabe dosisrelevanter Parameter wie z. B. CTDIVol bei CT-Untersuchungen verzichtet. Abschnittsweise kann auf Informationen im Prüfplan verwiesen werden oder eine direkte Übernahme per „Copy-&-Paste“-Funktion erfolgen.

Die bisher schon größtenteils in englischer Sprache mögliche Einreichung des medizinisch-wissenschaftlichen Formblatts wird nun auf sämtliche einzureichende Unterlagen ausgeweitet.

Für die Anzeigeverfahren nach StrlSchG sind die erforderlichen Informationen auf den Internetseiten des BfArM und AKEK zu finden [11, 12].

## Fazit

Die Änderung des StrlSchG durch das neue MFG eröffnet grundsätzlich ein neues Potenzial, die Attraktivität des Forschungsstandorts Deutschland zu stärken. Aus Sicht des Strahlenschutzes bleibt die Aufrechterhaltung eines hohen Schutzniveaus für Studienteilnehmende dabei weiterhin erhalten.

Aus Sicht der Forschenden bleibt die Frage, wie gut insbesondere die digitale Umsetzung gelingen wird und wie sich auch im Umgang mit diagnostischen Strahlenanwendungen außerhalb vom AMG und MPDG („freie“ oder „Non/non“-Studien) die Änderungen auswirken werden. Insbesondere der Single-Gate-Ansatz wird sich bewähren müssen, da er technische Neuerungen für alle Beteiligten bringen wird. Im Bereich der Strahlenforschung bestehen weiterhin gewisse Bedenken, dass die Anstrengungen zur Umsetzung der neuen Regelungen im Pharmabereich gegebenenfalls zu einer Benachteiligung anderer Forschungsbereiche führen könnten. Bisher scheinen jedoch alle Bereiche von den positiven Entwicklungen zu profitieren. Die mannigfaltigen und im Zusammenspiel durchaus komplexen Änderungen werden sich naturgemäß erst in den Jahren nach ihrer nun erfolgenden Umsetzung praktisch voll bewähren können.

DEGRO und BfS werden daher den im Rahmen der Umsetzung der Neuregelungen des MFG etablierten/bewährten Dialog weiterführen und ihre Beobachtungen und Erfahrungen mit den neuen Verfahren regelmäßig austauschen. Dadurch können diese kontinuierlich bewertet und optimiert werden, um die Ziele des MFG für alle Seiten zufriedenstellend umzusetzen.
